# Transcriptome-Wide Analysis of RNA m^6^A Methylation and Gene Expression Changes Among Two *Arabidopsis* Ecotypes and Their Reciprocal Hybrids

**DOI:** 10.3389/fpls.2021.685189

**Published:** 2021-06-10

**Authors:** Zhihui Xu, Xiaobo Shi, Mengmei Bao, Xiaoqian Song, Yuxia Zhang, Haiyan Wang, Hairong Xie, Fei Mao, Shuai Wang, Hongmei Jin, Suomeng Dong, Feng Zhang, Zhe Wu, Yufeng Wu

**Affiliations:** ^1^College of Life Science, Nanjing Agricultural University, Nanjing, China; ^2^State Key Laboratory for Crop Genetics and Germplasm Enhancement, Jiangsu Key Laboratory for Information Agriculture, Bioinformatics Center, Academy for Advanced Interdisciplinary Studies, Nanjing Agricultural University, Nanjing, China; ^3^Institute of Agricultural Resources and Environment, Jiangsu Academy of Agricultural Sciences, Nanjing, China; ^4^College of Plant Protection, Nanjing Agricultural University, Nanjing, China; ^5^Department of Biology, SUSTech-PKU Institute of Plant and Food Science, Southern University of Science and Technology, Shenzhen, China

**Keywords:** RNA m^6^A methylation, hybrid, heterosis, *Arabidopsis*, RNA modification dynamics

## Abstract

The remodeling of transcriptome, epigenome, proteome, and metabolome in hybrids plays an important role in heterosis. N(6)-methyladenosine (m^6^A) methylation is the most abundant type of post-transcriptional modification for mRNAs, but the pattern of inheritance from parents to hybrids and potential impact on heterosis are largely unknown. We constructed transcriptome-wide mRNA m^6^A methylation maps of *Arabidopsis thaliana* Col-0 and *Landsberg erecta* (Ler) and their reciprocal F_1_ hybrids. Generally, the transcriptome-wide pattern of m^6^A methylation tends to be conserved between accessions. Approximately 74% of m^6^A methylation peaks are consistent between the parents and hybrids, indicating that a majority of the m^6^A methylation is maintained after hybridization. We found a significant association between differential expression and differential m^6^A modification, and between non-additive expression and non-additive methylation on the same gene. The overall RNA m^6^A level between Col-0 and Ler is clearly different but tended to disappear at the allelic sites in the hybrids. Interestingly, many enriched biological functions of genes with differential m^6^A modification between parents and hybrids are also conserved, including many heterosis-related genes involved in biosynthetic processes of starch. Collectively, our study revealed the overall pattern of inheritance of mRNA m^6^A modifications from parents to hybrids and a potential new layer of regulatory mechanisms related to heterosis formation.

## Significance Statement

The reprogramming and corresponding effect of mRNA m^6^A methylation on hybrids remain highly unknown. We demonstrated the pattern of conserved inheritance of m^6^A methylation from parents to hybrids and the potential impact on heterosis formation, uncovering mRNA m^6^A methylation as a new layer of regulatory mechanisms in the formation of hybrid vigor.

## Introduction

Heterosis refers to the increased performance of hybrid offspring relative to their parents in many traits, such as growth rate and biomass (Birchler et al., 2003, 2010; Hochholdinger and Hoecker, 2007; Chen, 2010; Birchler, 2015). Both genetic and epigenetic mechanisms are thought to be involved in heterosis (Chen, 2013). Epigenetic changes have been found to impact hybrid vigor (Cubas et al., 1999; Manning et al., 2006; Shindo et al., 2006; Ni et al., 2009; He et al., 2010). DNA methylation level is altered by trans-chromosomal methylation (TCM) and trans-chromosomal demethylation (TCdM) (Greaves et al., 2014), which changes the overall DNA methylation level in the F_1_ hybrids, especially in regions that are differentially methylated in two parents (Shen et al., 2012). Histone modification patterns in hybrids of rice or maize have shown correlations between altered gene expression and changes in histone marks compared with the parents (He et al., 2010, 2013; Lv et al., 2019). In *Arabidopsis* hybrids, global histone modifications of the parents are largely transmitted to the F_1_ generation (Moghaddam et al., 2011; Dong et al., 2012; Yang et al., 2016). DNA methylation and histone modifications are altered at many loci, such as circadian clock associated1 (CCA1) and late elongated hypocotyl (LHY), which are associated with growth vigor in *Arabidopsis* F_1_ hybrids (Ni et al., 2009; Shen et al., 2012).

Recently, chemical modifications of mRNAs, such as N(6)-methyladenosine (m^6^A), N(1)-methyladenosine (m^1^A), and 5-methylcytosine (m^5^C), have emerged as an additional level of transcript regulation (Dominissini et al., 2012, 2016; Meyer et al., 2012; Li et al., 2016, 2017). m^6^A methylation is the most abundant type of modification for mRNAs, occurring in more than one-third of mammalian transcripts and half of the plant transcripts (Dominissini et al., 2012; Meyer et al., 2012; Li et al., 2014; Luo et al., 2014; Luo et al., 2020; Wan et al., 2015; Zhou et al., 2019; Miao et al., 2020). The m^6^A modification is reversible and dynamic, with m^6^A demethylase acting as an eraser and methyltransferase acting as a writer (Jia et al., 2011; Meyer and Jaffrey, 2017). Recognition of these dynamic m^6^A modifications by YTH domain-containing proteins leads to a broad range of functions associated with the change in mRNA stability, cap-independent translation, splicing, translation efficiency, and mRNA structure (Dominissini et al., 2012; Meyer et al., 2015; Meyer and Jaffrey, 2017), but the location of m^6^A in mRNA determines different functions (Gilbert et al., 2016). In the 5' UTR, m^6^A participates in mRNA cap-independent translation by directly binding to eukaryotic initiation factor 3 (eIF3) and then recruiting the 40S ribosomal subunit to initiate translation (Meyer et al., 2015). On the other hand, m^6^A in the 3' UTR has been reported to have several functions, such as promoting translation by binding with METTL3 and eIF3h to facilitate formation of the translation loop (Choe et al., 2018), regulating mRNA lifetime by binding with YTHDF2, which relocates transcripts to the P-body (Wang et al., 2014), and changing mRNA structure to affect RNA-protein interactions (Liu et al., 2015).

In *Arabidopsis thaliana*, m^6^A is essential in embryo development (Zhong et al., 2008). Further research revealed that m^6^A is also essential in post-embryonic development (Bodi et al., 2012), for example, for normal trichome morphology and correct timing of leaf formation (Arribas-Hernandez et al., 2018; Scutenaire et al., 2018; Wei et al., 2018), partly because it regulates the expression of key shoot meristem genes to control shoot apical meristem (SAM) proliferation (Shen et al., 2016). Transcriptome-wide mapping of m^6^A in *Arabidopsis* wild-type (WT) and related mutants indicated a complex relationship between m^6^A modifications and gene expression. Lack of FKBP12 interacting protein 37 (FIP37), a component of the methyltransferase complex in *Arabidopsis*, results in a dramatically reduced abundance of m^6^A, as most transcripts bearing m^6^A in WT are decreased in the mutant (Shen et al., 2016). In addition, further study showed that m^6^A inhibits mRNA degradation through inhibition of site-specific cleavage (Anderson et al., 2018). Nevertheless, it was reported that the highly expressed transcripts had fewer m^6^A modifications, as revealed by transcriptome-wide m^6^A patterns in *Arabidopsis* (Wan et al., 2015). Although m^6^A abundance varies among *Arabidopsis* accessions and affects transcript abundance, how m^6^A changes in F_1_ hybrids relative to their parents and its potential role in determining F_1_ hybrid vigor have not been clarified.

In this study, we selected two *Arabidopsis* ecotypes, namely, Col-0 and *Landsberg erecta* (Ler), and their F_1_ reciprocal hybrids, to investigate the potential effect of m^6^A on heterosis. We identified the distribution pattern and the intensity change in m^6^A in Col-0, Ler, and their F_1_ reciprocal hybrids. We showed that the peaks and distribution features of m^6^A methylation are highly conserved between accessions. Although changes in m^6^A intensity and transcript abundance within accessions are weakly positively correlated, upregulation of m^6^A between accessions tends to be associated with a downregulated abundance of mRNA and vice versa. We found that the overall m^6^A difference between the parents is attenuated at allelic sites in the hybrids, and that there is a negative correlation between the expression and corresponding m^6^A intensity of allelic genes. Interestingly, even though hundreds of m^6^A peaks are changed between the parents and hybrids, many biological functions of the corresponding genes are consistently affected, including the biosynthetic processes of starch, which have been reported to be associated with growth vigor. The data, therefore, suggest the overall pattern of mRNA m^6^A remodeling in hybrids, which may contribute to heterosis formation.

## Results

### Transcriptome-Wide Profile of m^6^A Methylation Among Col-0, Ler, and Their F_1_ Reciprocal Hybrids

**Figure 1:**
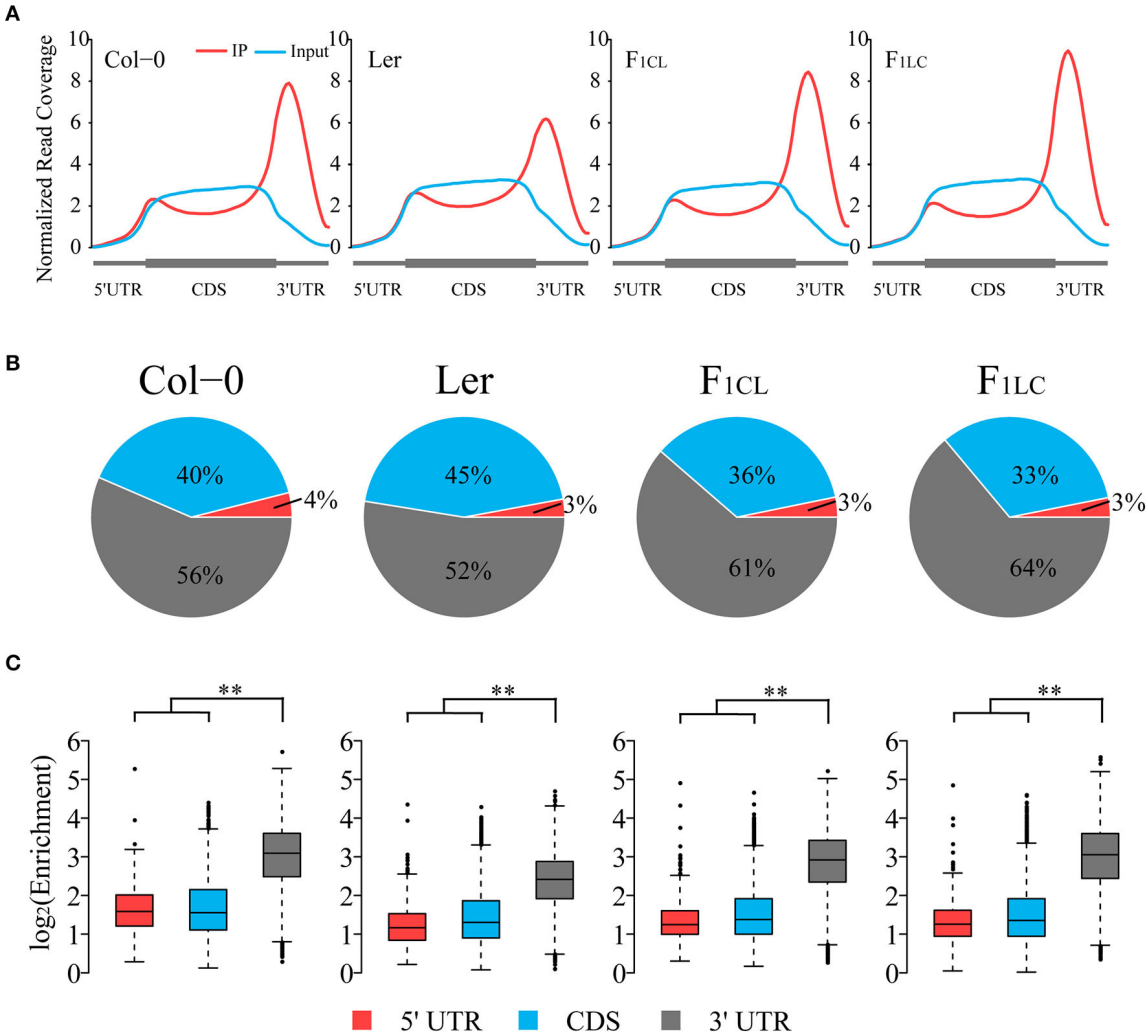
Global pattern of m^6^A peaks in Col-0, Ler, and their F_1_ reciprocal hybrids. **(A)** Coverage of normalized reads along transcripts. Each transcript is divided into three non-overlapping features: 5' UTR, CDS, and 3' UTR. **(B)** Distribution of m^6^A peaks in transcript features of parents and hybrids. **(C)** Relative enrichment of m^6^A peaks of each transcript feature. Enrichment = Normalized m^6^A-seq reads divided by normalized input reads of each peak. **p < 2.2e−16, Wilcoxon rank-sum test.

To explore RNA m^6^A abundance variation between the two ecotypes and its alteration in hybrids, we first analyzed transcriptome-wide m^6^A profiles among Col-0, Ler, and their F_1_ reciprocal hybrids (Supplementary Figure 1A) by applying N^6^-methyladenosine sequencing (m^6^A-seq) with two biological replicates. Sequencing data of RNA input and immunoprecipitation (RIP) are highly correlated between replicates, indicating the high quality of m^6^A-seq in this study (Supplementary Figures 1B,C). We found that the normalized reads from m^6^A-RIP of all samples are enriched in the 3' UTR of the transcripts (Figure 1A), which is similar to the results of previous research (Luo et al., 2014; Wan et al., 2015). The normalized read depth in Ler is significantly lower than that of the other three samples, suggesting that the overall m^6^A abundance of Ler was lower (Figure 1A). To exclude the possible bias introduced by the reference genome, we performed exact analysis using the Ler reference genome rather than Col-0 and still obtained identical results (Supplementary Figure 2). Interestingly, we did not find low m^6^A abundance in the 3' UTR of the two hybrids, similar to Ler.

To further study global patterns of m^6^A in Col-0, Ler and their hybrids, we identified m^6^A peaks using a transcriptomic peak caller, METPeak (Cui et al., 2016). A total of 13,145, 13,562, 12,956, and 12,542 peaks are detected in Col-0, Ler, F_1CL_, and F_1LC_, respectively (Supplementary Table 1); and these peaks were located in ~9,778, 9,920, 10,066, and 10,017 protein-coding genes, respectively. The majority of these genes have one or two m^6^A sites (Supplementary Figure 3A), which is consistent with a previous report (Wan et al., 2015). In agreement with the distribution of m^6^A-seq reads, the majority of the m^6^A peaks are enriched in the 3' UTR and CDS region, while only 3–4% of the m^6^A peaks are located in the 5' UTR (Figure 1B). The enrichment degree of peaks in the 3' UTR is significantly higher (Wilcoxon rank sum test, *p* < 2.2e−16) than that of peaks in the 5' UTR and CDS among the four samples (Figure 1C). As expected, we also found that the enrichment of the m^6^A peaks in Ler is significantly lower than that in the other groups (Wilcoxon rank sum test, *p* < 2.2e−16, Supplementary Figure 3B).

To further analyze the feature of the distribution of m^6^A peaks, we counted the number of peaks around the start codon segment and the stop codon segment (200 nt centered on the start codon and stop codon, respectively), and found that ~40% of the peaks are located in these two regions (Supplementary Figure 3C). The number of peaks in the start codon is relatively low in all four samples. However, there are more than 4,000 m^6^A peaks located in the stop codon segment (Supplementary Figure 3C), which is consistent with previous findings in mammals and plants showing that m^6^A peaks are preferentially located around stop codons (Dominissini et al., 2012; Luo et al., 2014; Wan et al., 2015).

### Variations of m^6^A Modification Among the Parental Lines and Hybrids

**Figure 2:**
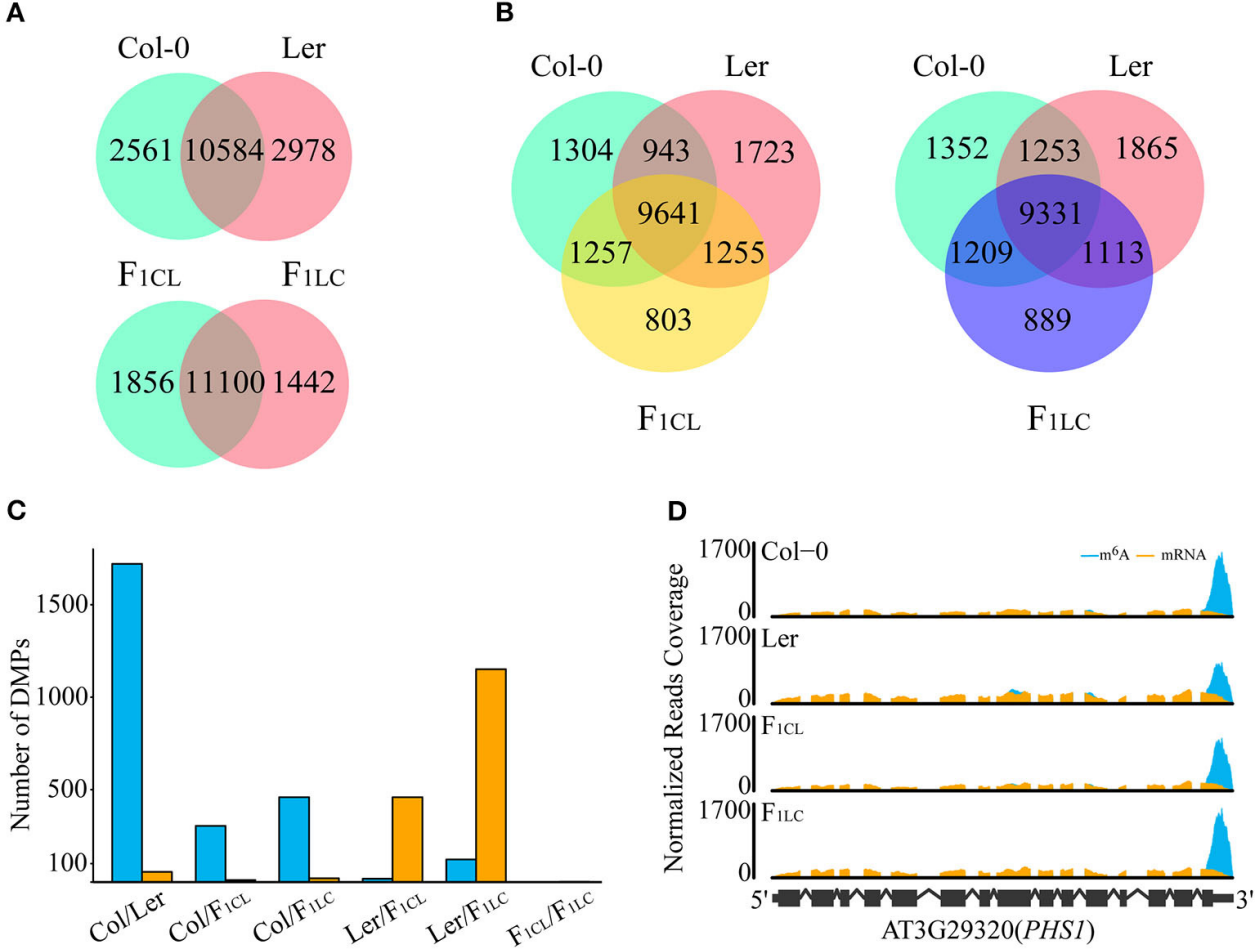
Differences in m^6^A modifications among Col-0, Ler, and their F_1_ reciprocal hybrids. **(A,B)** Number of shared m^6^A peaks between accessions. **(C)** Number of DMPs in each comparison. Blue bars, DMPs showing upregulated m^6^A intensity in the former comparison. Orange bars, DMPs showing upregulated m^6^A intensity in the latter. **(D)** Diagram for differentially methylated peaks. m^6^A, normalized IP reads; mRNA, normalized input reads.

Previous research has shown that m^6^A is highly conserved between two accessions of *Arabidopsis*, namely, Can-0 and Hen-16 (Luo et al., 2014). We found that 10,584 m^6^A peaks (80.5% of Col-0, 78% of Ler; Figure 2A) are common between Col-0 and Ler, and that these peaks are located in 8,302 expressed transcripts (49.4% of the total). In addition, we found that the majority of the m^6^A peaks are common among the parental lines and F_1_ hybrids. There are 9,641 (74.4% of F_1CL_) and 9,331 (74.4% of F_1LC_) m^6^A peaks that are common between the parents and the F_1_ hybrids, respectively (Figure 2B). These peaks are also located in 7,844 and 7,723 of the expressed transcripts in F_1CL_ and F_1LC_, respectively (Supplementary Figure 3E). The common peaks (11,000) between F_1CL_ and F_1LC_ account for 85.6–88.5% of the total peaks in F_1_ hybrids (Figure 2A), which is slightly higher than that in the two parents. Collectively, these data indicate a more general conservation pattern of RNA m^6^A modification among accessions and hybrids of *Arabidopsis*.

Considering the obvious difference in m^6^A levels between Col-0 and Ler, it is necessary to determine whether common m^6^A peaks between any two samples are significant differentially methylated peaks (DMPs). We established two criteria for DMPs: (1) passed Fisher's exact test after multiple comparison corrections (FDR < 0.05); (2) the difference in peak enrichment between any two samples was larger than a 1.5-fold change. Eventually, we identified 1,776 DMPs (16.8% of the common peaks) between the parents, among which the intensity of 1,721 (16.3%) peaks, as expected, is higher in Col-0 (Figures 2C,D, Supplementary Table 2). For the comparison between F_1_ reciprocal hybrids, we found only 2 DMPs (0.02%), suggesting that paternal or maternal effects on the level of m^6^A modifications are weak in *Arabidopsis* (Figure 2C). For the m^6^A peaks shared between the parents and F_1CL_ or between the parents and F_1LC_, we identified 315, 479, 477, and 1,273 DMPs, respectively (Figure 2C). Taken together, the intensity of common m^6^A peaks tends to be conserved between accessions or during inheritance from parents to hybrids.

### Relationship Between Transcript Abundance and m^6^A Modification Level

**Figure 3:**
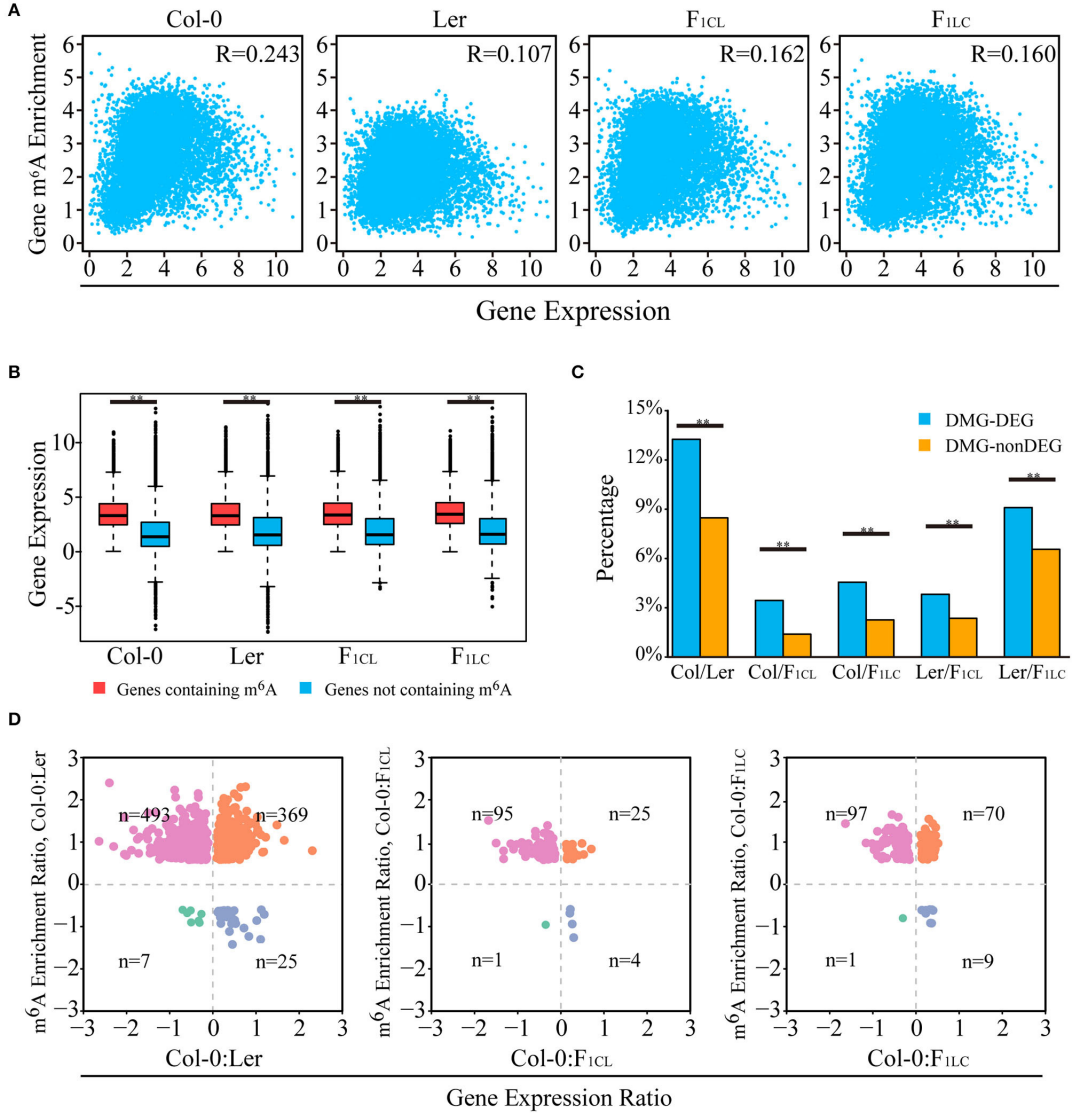
Relationship between m^6^A methylation level and transcript abundance. **(A)** Scatter plot showing the correlation of m^6^A modification and transcript abundance. R indicates Pearson correlation coefficient. **(B)** Transcripts with m^6^A peaks showing higher abundance levels. **(C)** Percentage of DEGs (differentially expressed genes) overlapping with DMGs (genes with differentially m^6^A-methylated peaks), indicated as DMG-DEGs, and percentage of non-DEGs associated with DMGs, indicated as DMG-nonDEGs. **p < 1e−6, Wilcoxon rank-sum test. **(D)** Scatter plot of DMG-DEGs between accessions showing the relationship of m^6^A modification and transcript abundance. For example, the m^6^A enrichment ratio of Col-0: Ler is calculated as log2 (enrichment of Col-0/enrichment of Ler) of m^6^A peaks. The gene expression ratio of Col-0: Ler is calculated as log2 (FPKM of Col-0/FPKM of Ler) of transcripts. n indicates number of DMG-DEGs in each quadrant. For **(A–D)**, gene m^6^A enrichment is calculated by normalized m^6^A-seq reads number divided by normalized input reads of peaks within the transcript, and gene expression is indicated by the FPKM of the input RNA-seq data.

Multiple recent studies have indicated complex functions of m^6^A in transcription regulation with the ability to stabilize (Luo et al., 2014; Anderson et al., 2018) or destabilize mRNAs in *Arabidopsis* (Wan et al., 2015). We analyzed the relationships of transcript abundance and the corresponding m^6^A levels. We found a weak positive correlation between the expression abundance and intensity of m^6^A modification on one gene within each accession (Figure 3A). Overall, the genes with m^6^A modification show significantly higher expression than non-m^6^A-containing genes (Figure 3B). In addition, more than 60% of the expressed genes are associated with at least one m^6^A peak (Supplementary Figure 3A, Supplementary Table 1).

Next, we investigated the relationship between changes in m^6^A methylation and transcript abundance in the parent lines and their F_1_ reciprocal hybrids. We first identified differentially expressed genes (DEGs) between the lines (Supplementary Figures 4A,B) and checked the overlap between DEGs and DMPs. We found that the proportion of DEGs associated with DMPs is significantly higher than that of non-DEGs (Figure 3C). Even so, only 3.29–13.26% of the DEGs are associated with DMPs between the parent lines and hybrids (Figure 3C). Taken together, these results indicated that changes in m^6^A intensity on transcripts tend to be associated with changes in abundance, and that most DEGs are not directly associated with m^6^A changes in *Arabidopsis*.

We then focused on genes with significant changes in both expression and m^6^A modification between accessions. Most DMPs showed upregulated m^6^A intensity in Col-0 between Col-0/Ler (comparison between Col-0 and Ler), as well as between Col-0/F_1CL_ and between Col-0/F_1LC_ (Figure 3D). A total of 862 DEGs between Col-0/Ler are associated with DMPs upregulated in Col-0, among which there are significantly more downregulated expressed genes than upregulated genes in Col-0 (Figure 3D, *p* = 2.41e−5, chi-square test). A similar pattern is also found in Col-0/F_1CL_ (*p* = 1.7e−10) and Col-0/F_1LC_ (*p* = 0.037, Figure 3D). There are more DMPs showing downregulated m^6^A intensity in Ler between Ler/F_1CL_ and between Ler/F_1LC_, and these DMPs are also associated with more genes with upregulated expression in Ler (Supplementary Figure 4C, *p* = 1.19e−12 for Ler/F_1CL_; *p* = 2.38e−20 for Ler/F_1LC_). This result indicates that downregulated DMPs tend to be associated with more upregulated DEGs and vice versa between accessions of *Arabidopsis*, implying that the complexity of m^6^A function affects the transcript abundanceof genes.

### Relationship Between Non-additive Expression and Non-additive m^6^A Modification

We identified 2,758 and 4,123 genes showing non-additive expression in F_1CL_ and F_1LC_, respectively. Similar to gene expression, the inheritance of m^6^A modifications in hybrids can be additive or non-additive. We defined m^6^A peaks with a significant change between enrichment value in hybrid and the average enrichment value of parents (MPV) (FDR < 0.05, see methods for detail) as non-additive m^6^A modified peaks. The majority (95.6 and 95.2%) of the m^6^A peaks show additive patterns in both hybrids, while only 538 and 563 peaks in F_1CL_ and F_1LC_, respectively, are non-additive (Supplementary Table 3). Moreover, non-additive m^6^A peaks are significantly associated with non-additively expressed genes in both hybrids (Supplementary Table 3, *p* < 2.2e−16 for both F_1CL_ and F_1LC_, chi-square test). We still observed that only 6.53–6.82% of non-additively expressed genes show a non-additive pattern of m^6^A modification, indicating that m^6^A may play a role in the regulation of non-additive gene expression in *Arabidopsis* hybrids.

### Relationship Between Allelic Gene Expression and Allelic m^6^A Methylation in F_1_ Hybrids

**Figure 4:**
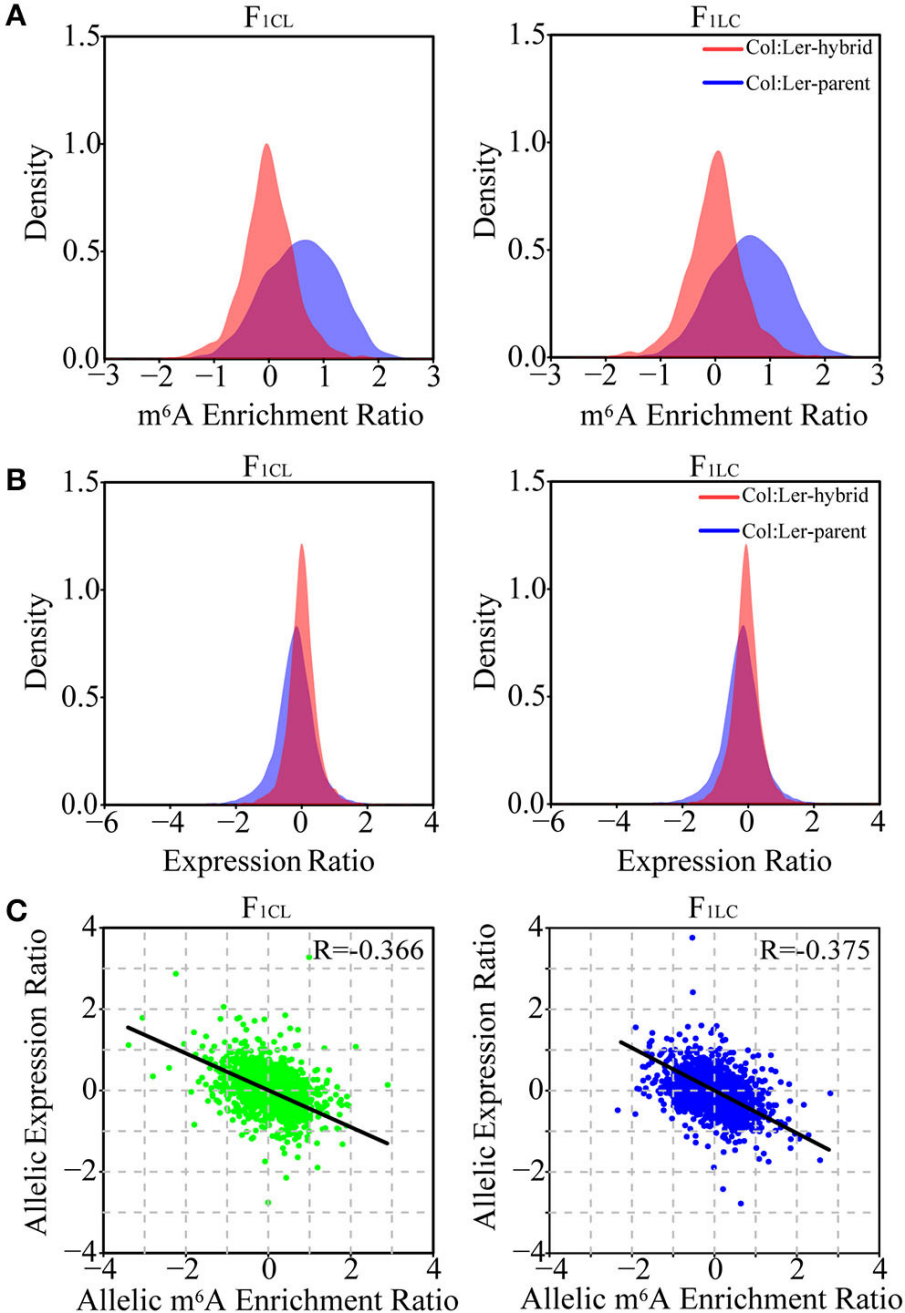
Relationship between allelic gene expression and allelic m^6^A methylation in F_1_ hybrids. Density distribution of the **(A)** m^6^A enrichment ratio and **(B)** gene expression ratio between allelic sites of Col-0 and Ler (marked as Col: Ler-parent) and the ratio between allelic sites inherited from Col-0 and Ler in the hybrids (Col: Ler-hybrid). **(C)** Scatter plot showing the correlation between allele-specific expression and allele-specific m^6^A methylation in the hybrids. Only the reads of m^6^A-seq or RNA-seq mapped to the SNPs between Col-0 and Ler with high confidence are used in the analysis.

To analyze the allelic bias in gene expression and m^6^A modifications in hybrids, we identified single-nucleotide polymorphisms (SNPs) between Col-0 and Ler with stringent criteria (see methods) and used these SNPs to determine the reads of RNA-seq or m^6^A-seq generated from the allele of Col-0 or Ler. A total of 76,983 SNPs with high confidence are identified. These SNPs associate with 8,972 and 8,991 genes and with 2,509 and 2,325 m^6^A peaks in F_1CL_ and F_1LC_, respectively, which are used in the following analysis. As expected, we still observed significantly higher m^6^A modification in Col-0 than in Ler on these SNPs (Figure 4A). Nevertheless, this bias tends to disappear between the two parental alleles in the hybrids. The log-transformed mean value of the m^6^A ratio between the two allelic SNPs is close to zero, and the majority of the ratio (94.1% for F_1CL_ and 93.6% for F_1LC_) falls within the interval (−1, 1) in both hybrids (Figure 4A), indicating that the overall m^6^A difference between the parents is attenuated at allelic sites in the hybrids. The pattern of attenuation is not observed for the expression of allelic genes (Figure 4B). We have identified only four and seven peaks showing significant allele-specific RNA m^6^A methylation (FDR < 0.05, see Methods) in the hybrids, implying extremely rare allele bias of RNA m^6^A methylation after the combination of the two parental genomes. Despite the smaller difference in m^6^A abundance between the alleles, the correlation between the allelic abundance of mRNA and the corresponding allelic intensity of m^6^A methylation is negative (Figure 4C). This result is consistent with the relationship between DEGs and DMPs.

### Biological Function of Genes Associated With Significant Changes in m^6^A

**Figure 5:**
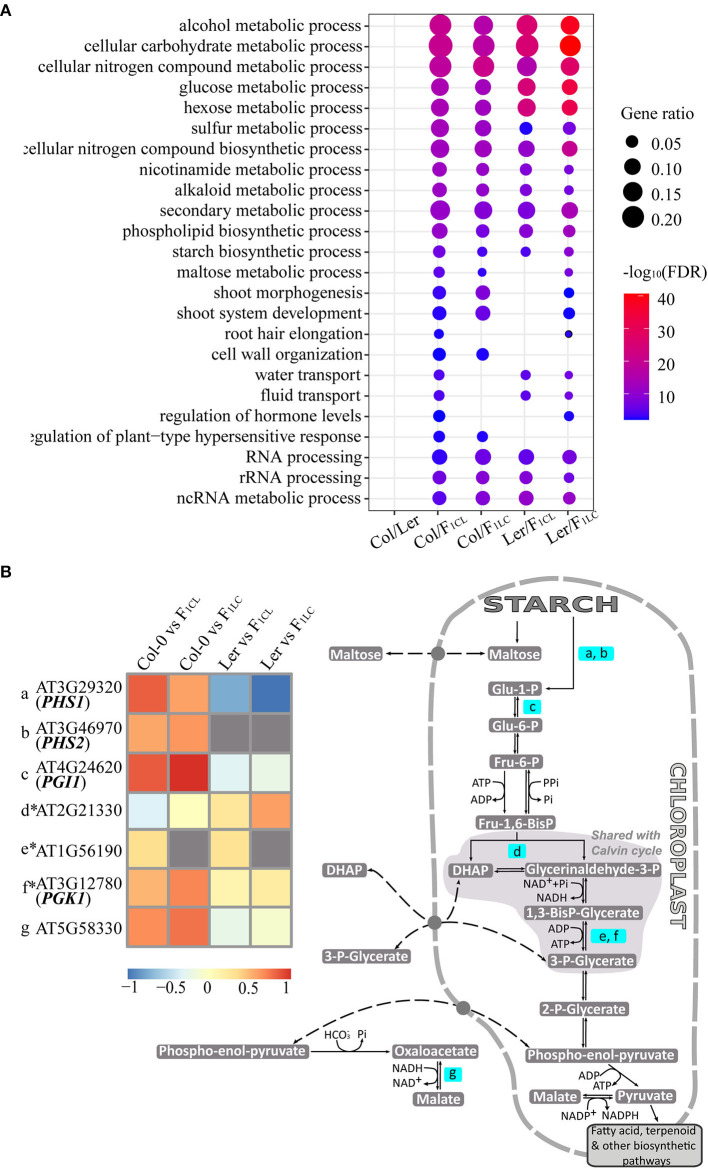
Enriched biological functions of differentially m^6^A-methylated genes. **(A)** GO terms of genes associated with DMPs that are enriched only in comparisons of parents/hybrids, e.g., m^6^A-methylated peaks showing significant differences between Col-0 and F_1CL_. Only some enriched GO terms are shown in the figure, and all GO terms are listed in Supplementary Table 4. **(B)** A diagram for genes with DMPs involved in starch biosynthetic process related to biomass in *Arabidopsis*. The pathway is modified based on the Mapman database. Only genes in the pathway with DMPs are highlighted, of which m^6^A methylation differences between parents/hybrids are shown in the heatmap. *indicates the genes showing non-additive m^6^A methylation in the hybrids simultaneously.

F_1_ hybrids crossed by ecotypes of *Arabidopsis*, as well as Col-0 × Ler (Groszmann et al., 2014), showed clear growth vigor (Supplementary Figure 1), but the relationship between heterosis and changes in m^6^A abundance between the parent lines and hybrids was unknown. We first focused on the function of genes showing significantly differential m^6^A methylation (Supplementary Table 4), which were referred to as differentially m^6^A-modified genes (DMGs). We identified 462 enriched GO terms of DMGs between Col-0 and Ler, among which 160–294 (34–63%) are also identified as enriched GO terms of DMGs generated from the comparisons between the parents and hybrids (Supplementary Table 5). Interestingly, the enriched GO terms of DMGs between the parents and hybrids tend to be consistent. For instance, we found 319 enriched GO terms of DMGs between Col-0/F_1CL_, among which 231–267 (72–84%) are also identified in the Col-0/F_1LC_, Ler/F_1CL_, and Ler/F_1LC_ comparisons. These data implied that there is clear heterogeneity of biological functions affected by differential m^6^A modification between Col/Ler (between-parent difference) and between parent/hybrid (parent-hybrid difference). We kept the enriched GO terms of DMGs from the parent-hybrid comparison but not from the between-parent comparison and found a clear trend of enriched biological functions, such as biosynthetic and metabolic processes of multiple carbohydrates, secondary metabolic processes, and development of shoot, root hair, and so on (Figure 5A, Supplementary Table 4), among which starch biosynthetic process was reported to be involved in heterosis (Chen, 2013).

Hybrid vigor has been found to be related to changes in transcription, epigenetic modifications, and protein abundance (Chen, 2013). Considering that m^6^A is involved in multiple biological processes related to RNA fate at the post-transcriptional level, it is worthwhile to focus on the function of genes showing differential m^6^A modification without changes in gene expression. We found that the enriched GO terms of DMGs and not DEGs between parents/hybrids are associated with membrane- or chloroplast-located proteins, transport, or the proteasome complex (Supplementary Figure 5A). This pattern is clearly different from the enriched GO terms of the DEGs but not DMGs between parent/hybrid, which are associated with stress response genes, mitochondria-located genes, etc (Supplementary Figure 5B). These data implied that m^6^A modification could be involved in the formation of F_1_ hybrid vigor through post-transcriptional regulation of mRNA without changing the abundance.

Several genes involved in starch and carbohydrate metabolism promote growth and biomass vigor in *Arabidopsis* (Chen, 2013), so we focused on the DMGs involved in the starch biosynthetic process (Figure 5B). There are 51 DMGs from all four parent/hybrid comparisons annotated as genes of the starch biosynthetic process (GO: 0019252). We checked the published biological functions against the TAIR database one by one and found 20 genes associated with the biomass and growth rate of *Arabidopsis* (Supplementary Table 6). We visualized seven genes of 51 DMGs that are annotated as starch metabolism genes through Mapman (Thimm et al., 2004). Interestingly, six genes are located in chloroplasts and involved in the same pathway, and four of them control biomass and growth rate in *Arabidopsis* based on published results (Figure 5B). Collectively, these data indicated the strong association between changes in m^6^A methylation and the growth vigor of F_1_ hybrids.

## Discussion

Multiple transcriptome-wide maps revealed highly conserved patterns of m^6^A methylation among *Arabidopsis* accessions (Can-0 and Hen-16) or organs (leaf, root, and flower) (Luo et al., 2014; Wan et al., 2015). More than 70% of m^6^A peaks are shared between *Arabidopsis* Can-0 and Hen-16 (Luo et al., 2014), similar to the percentage (78%) of peaks shared between Col-0 and Ler in this study. We also found that ~74% of m^6^A peaks are shared between the parents and hybrids. In addition, our results indicated that m^6^A modifications in hybrids are enriched around the 3' UTR, stop and start codons of transcripts, showing consistent features across accessions and organs (Luo et al., 2014; Wan et al., 2015). Moreover, RNA m^6^A methylation peaks are also conserved between two inbred lines (B73 and Mo17) of maize (Luo et al., 2020) and two tissues of rice (Li et al., 2014). In summary, these results implied a more general conservation pattern of m^6^A methylation in plants, which could be related to the fundamental role of m^6^A methylation in plant development (Zhong et al., 2008; Shen et al., 2016; Anderson et al., 2018). Nevertheless, a recently published study showed that there are much more genes with differentially m^6^A level or non-additively m^6^A variation in maize hybrid (B73 × Mo17) compared with the parents (Luo et al., 2021), implying that the pattern of m^6^A reprogramming in hybrid is related to species or parent lines with different degree of variation.

The effects of m^6^A modification on gene expression vary among genes. In an *Arabidopsis* demethylase ALKBH10B loss-of-function mutant, mRNAs of flower development genes, such as FT, SPL3, and SPL9 show increased m^6^A modification but reduced stability (Duan et al., 2017). Nevertheless, lack of m^6^A modifications on the mRNA of the WUS and STM genes enhances their stability in the FIP37 mutant line of *Arabidopsis* (Shen et al., 2016). Additional studies have indicated the biological functions of stabilizing or destabilizing mRNAs in *Arabidopsis* (Luo et al., 2014; Wan et al., 2015; Anderson et al., 2018). The data also indicated the conflicting functions of m^6^A in regulating gene expression. Overall, we observed a very weak positive correlation between the abundance of mRNA and the intensity of m^6^A modification within each of the accessions. However, we also found that mRNAs with significantly decreased methylation of m^6^A tend to show upregulated expression between accessions or between parents and hybrids. The complex regulatory roles of m^6^A in transcript abundance might be correlated with its location (Luo et al., 2014), differences between readers (Wei et al., 2018), or the RNA structure dependent on m^6^A (Liu et al., 2015).

The molecular mechanism of heterosis is quite complex; and omics methods, ranging from transcriptomics to metabolomics, have provided novel insights into the mechanism (Chen, 2013). Changes in epigenetic modifications, such as histone methylation in hybrids, could promote growth by altering gene expression (Ni et al., 2009). As a newly identified reversible modification of RNA, the reprogramming of m^6^A in hybrids and the corresponding functions related to heterosis remain largely elusive. The data indicated that most of the differentially expressed genes are not associated with differential m^6^A methylation, and that only a few hundred m^6^A peaks are significantly changed between parents and hybrids. However, these peaks are associated with many biological functions, of which 20 of 51 starch- and carbohydrate-related genes are confirmed as being associated with biomass vigor in *Arabidopsis* (Supplementary Table 6). We did not identify the genes showing differential m^6^A methylation involved in the circadian rhythm regulatory network, for instance, LHY, GI, CCA1, and TOC1, which are also related to biomass vigor in hybrids crossed by two accessions, namely, Col-0 and C24 (Chen, 2013). We propose two possible reasons. One could be that the different molecular bases of heterosis between F_1_ hybrids are crossed by different ecotypes. The hybrids of C24 × Col and Col × Ler showed differences in growth vigor at various time points of vegetative development (Groszmann et al., 2014). Another reason could be that circadian rhythm-related genes tend to promote growth through the regulation of transcription. We found that some circadian genes, such as GI and TOC1, are differentially expressed between the parents and hybrids, while a considerable number of the 20 genes involved in the starch biosynthetic process showed only differential m^6^A methylation rather than differential expression (Supplementary Table 7). Since m^6^A controls RNA fate-related processes, such as mRNA stability, transport, or translation (Dominissini et al., 2012; Meyer et al., 2015; Meyer and Jaffrey, 2017), this study indicates a new layer of regulatory mechanisms contributing to heterosis at the post-transcriptional level in *Arabidopsis*.

### Experimental Procedures

#### Plant Materials and Growth Conditions

Plant materials included two *Arabidopsis* accessions (Col-0, Ler) and their F_1_ reciprocal hybrids. F_1_ seeds were produced by hand pollination between Col-0 and Ler. Seeds were sown on soil, stratified at 4°C for 3 days to synchronize germination. Plants were then shifted into greenhouse and grown under a long-day condition (16 h in light and 8 h in dark) at 22°C for 21 days. Above-ground tissues were harvested and stored at −80°C for the following experiments.

#### MeRIP Libraries Construction and Sequencing

MeRIP libraries preparation mainly followed a published procedure (Dominissini et al., 2013). Briefly, total RNA was extracted from leaves in 50 mL conicals using TRIzol (15596018, Ambion, Austin, TX, United States). Poly(A) RNA was enriched (MRN10, Sigma-Aldrich, Saint Louis, MO, United States) and fragmented into ~100 nt by fragmentation reagent (AM8740, Invitrogen, Carlsbad, CA, United States) for 15 min at 70°C. Few microliters of fragmented RNA was saved as input control, and the left was incubated with m^6^A antibody (202003, Synaptic Systems, Goettingen, Germany), in 1x IP buffer supplemented with RNasin Plus (N2611, Promega, Madison, WI, United States) for 4 h at 4°C. The antibody-bound RNA was then incubated with pre-blocked protein A beads (10001D, Invitrogen, Carlsbad, CA, United States) at 4°C for 2 h. The immunoprecipitated RNA was released using an elution buffer (1x IP buffer supplemented with 6.7 mM N^6^-methyladenosine, M2780, Sigma-Aldrich, Saint Louis, MO, United States). Input and IP libraries were constructed using NEBNext Ultra RNA Library Prep Kit for Illumina (E7645S, NEB, Ipswich, MA, United States) and subjected to sequencing on the Illumina Hiseq X-10 platform.

#### Reads Pre-processing and Alignment

Raw reads of input and IP samples were processed by trim-galore (version 0.4.1) to remove adaptors and low quality reads and then mapped to the *Arabidopsis* Col-0 reference genome (TAIR 10) using Tophat2 (version 2.1.1) (Kim et al., 2013) with Araport11 annotation in the analyses for parental lines and hybrid lines. We also used Ler reference genome and corresponding annotation (downloaded from NCBI, accession number GCA_001651475.1) to check for possible bias introduced by the reference genome. The parameters were modified (–read-edit-dist 5, –N 5) to obtain more SNP information of Ler and F_1_ hybrids. Multiple mapped reads were filtered using the SAMtools package (version 1.9) (Li et al., 2009). Only paired unique reads were used for downstream analysis.

#### N(6)-Methyladenosine Peak Identification and Annotation

MeTPeak (Cui et al., 2016), a transcriptomic peak caller, was used to identify m^6^A peaks. In order to get confidence peaks, we maintained peaks on genes with FPKM ≥ 1. Moreover, to avoid huge differences in the calculation of peak enrichment due to insufficient coverage, we performed a random sampling of genomic regions and calculated reads of all input samples, and high confidence peaks were selected if the peak region satisfied Input FPKM ≥ 5.

To define m^6^A peak summits, two repeats of input and IP sample were merged, and the coverage of each base of peaks was counted by in-house script (Supplementary Scripts 1–3). The residual was calculated by IP reads subtracted by input reads, and the point with the largest residual was referred to as peak summit. The peak summits were intersected with protein-coding gene sequences, which were integrated into a tiered order−3′UTR, 5′ UTR, and CDS, to determine their locations (Supplementary Script 4). Additionally, m^6^A peaks were assigned to start codon and stop codon segments, which was 200 nt centered to start codon and stop codon, respectively, to identify the preference of m^6^A peaks.

#### Identification of Differentially Methylated Peaks and Additive/Non-Additive Methylated Peaks

The common m^6^A peaks between any two samples were defined according to whether they intersected with each other. We calculated read counts of IP and input replicates for each m^6^A peak of every comparison group (Supplementary Script 5). A 2 × 2 contingency table was filled by IP and input normalized reads of samples, respectively. A Fisher's exact test was performed to identify m^6^A differentially methylated peaks, and *p*-value was adjusted by Bonferroni–Holm correction using R scripts. The differentially methylated peaks should satisfy two requirements: (1) padj <0.05; (2) the difference between any two samples >1.5.

To classify non-additive and additive methylated peaks, Fisher's exact test was performed by comparing the input and IP normalized reads of hybrid and the average of parents' input and IP normalized reads. Only common peaks with padj <0.05 were considered as non-additive methylated peaks. Otherwise, they were referred to as additively methylated peaks.

#### Identification of Differentially Expressed Genes and Additive/Non-Additive Expressed Genes

The number of reads for each gene was counted using HTSeq (Anders et al., 2015) with a default setting. R package DESeq2 (version 1.22.2) was used for analyzing differentially expressed genes, and only genes with padj <0.05 were considered as DEGs. If the expression of genes in hybrids was significantly different from mid-parent value (padj <0.05), these genes were classified as non-additive expressed genes, and the others were referred to as additive expressed genes.

#### Gene Ontology Analysis

The gene sets were submitted to agriGO database (Tian et al., 2017) to perform GO enrichment analysis. Functional enrichment was performed using the singular enrichment analysis (SEA) tool and TAIR genome locus (TAIR 10) as background. The GO terms with FDR ≤ 0.01 were considered to be enriched.

#### Analysis of Allelic Expression and Allelic N(6)-Methyladenosine Enrichment

To obtain confidence SNPs between Col-0 and Ler, the Ler (downloaded from NCBI) and Col-0 reference genomes (TAIR 10) were cut into 100 bp fragments with 1 bp shift, and then mutually mapped to the reference genome. The read counts of each position were called using the SAMtools “mpileup” command with the parameter “-f.” SNPs were first identified if site coverage ≥90X and mutant ratio (mutants/covered reads) ≥90%. The input and IP reads of F_1CL_ and F_1LC_ were separately mapped to the Col-0 reference and the Ler reference, and the reads covered SNPs were calculated. Theoretically, the reads mapped to the corresponding coordinate of the Col-0 and Ler references should be identical, or at least with small bias. Thus, SNPs with severe biased reads (the difference of reads mapped to the corresponding SNPs of two references was more than 10%) were excluded. Additionally, the SNPs that were not homozygous in parent lines were filtered. For allele-specific methylation analysis (Supplementary Script 6), we first calculated reads at SNPs within m^6^A peaks of IP and input replicates of F_1_ hybrids, and then filled a 2 × 2 contingency table with normalized reads. A Fisher's exact test was performed to identify allele-specific methylated peaks, and *p*-value was adjusted by Bonferroni-Holm correction using R scripts. Peaks with significant allelic methylation difference (FDR < 0.05) were identified as allele-specific peaks.

## Data Availability Statement

The original contributions presented in the study are publicly available. All the sequencing data have been deposited in The National Genomics Data Center (NGDC) under accession number CRA003884.

## Author Contributions

YW conceived the project. ZX, SD, FZ, and ZW designed the experiments. ZX, XBS, MB, YZ, HW, HX, SW, and HJ performed the experiments. ZX, XQS, FM, and YW conducted bioinformatics analyses. ZX, SW, and YW wrote the article. All authors contributed to the article and approved the submitted version.

## Conflict of Interest

The authors declare that the research was conducted in the absence of any commercial or financial relationships that could be construed as a potential conflict of interest.

## Supplementary Material

Supplementary Figure 1 Phenotypes of *Arabidopsis* lines and quality of sequencing data. **(A)** The biomass vigor of both F_1_ hybrids is higher than that of Col-0 and Ler. Scale bar = 10 mm. Spearman correlations between two biological replicates of input **(B)** mRNA-seq and **(C)** m^6^A-seq in Col-0, Ler, F_1CL_, and F_1LC._.

Supplementary Figure 2 Global pattern of m^6^A peaks using the Ler genome sequence as a reference. **(A)** Coverage of normalized reads along transcripts. Each transcript is divided into three non-overlapping features: 5' UTR, CDS, and 3' UTR. **(B)** Distribution of m^6^A peaks in transcript features of parents and hybrids. **(C)** Relative enrichment of m^6^A peaks of each transcript feature. Enrichment = Normalized m^6^A-seq read number divided by normalized input reads of each peak. ^**^*p* < 2.2e−16, Wilcoxon rank-sum test.

Supplementary Figure 3 Features of m^6^A modifications among the parent lines and F_1_ hybrids. **(A)** Number of peaks on transcripts. **(B)** Cumulative plot of m^6^A methylation enrichment in Col-0, Ler, F_1CL_, and F_1LC_. ^**^*p* < 2.2e−16, Wilcoxon rank-sum test. **(C)** Number of m^6^A peaks located at the start codon and stop codon of transcripts. **(D,E)** Number of shared genes containing m^6^A peaks between accessions.

Supplementary Figure 4 Diagram of the relationship between m^6^A methylation level and transcript abundance. **(A)** Number of DEGs between parents and hybrids. **(B)** Number of up- or downregulated DEGs in comparisons of parents/hybrids. **(C)** Scatter plot of DMG-DEGs between accessions showing the relationship of m^6^A modification and transcript abundance. DMG-DEG indicates DEGs overlapping with DMGs (genes with differentially m^6^A-methylated peaks). For example, the m^6^A enrichment ratio of Ler: F_1CL_ is calculated as log2 (enrichment of Ler/enrichment of F_1CL_) of m^6^A peaks. The gene expression ratio of Ler: F_1CL_ is calculated as log2 (FPKM of Ler/FPKM of F_1CL_) of transcripts. *n* indicates the number of DMG-DEGs in each quadrant. Gene m^6^A enrichment is calculated by normalized m^6^A-seq reads number divided by normalized input reads of peaks within the transcript, and gene expression is indicated by the FPKM of the input RNA-seq data.

Supplementary Figure 5 Enriched biological functions of differentially m^6^A-methylated genes. **(A)** Enriched GO terms of genes associated with differentially m^6^A-methylated peaks (DMPs) that are not DEGs in comparisons of parents/hybrids. **(B)** Enriched GO terms of DEGs not associated with differentially m^6^A-methylated peaks (DMPs) in comparisons of parents/hybrids. Only some of the enriched GO terms enriched in the comparisons between parents/hybrids rather than Col/Ler are shown in the figure. All the GO terms are listed in Supplementary Table 4.

Supplementary Table 1 List of m^6^A peaks identified in all the samples.

Supplementary Table 2 List of DMPs between the samples.

Supplementary Table 3 List of non-additive and additive expressed genes and m^6^A peaks in hybrids.

Supplementary Table 4 The results of GO analysis of DMPs in each comparison.

Supplementary Table 5 Shared GO terms of any two samples.

Supplementary Table 6 Genes related to growth vigor.

Supplementary Table 7 m^6^A methylation of starch biosynthetic process and circadian rhythm-related genes.
