# Supplementary figures and images for: Transcriptome-Wide Analysis of RNA m6A Methylation and Gene Expression Changes Among Two Arabidopsis Ecotypes and Their Reciprocal Hybrids
Source: Front Plant Sci. 2021 Jun 10;12:685189. doi: 10.3389/fpls.2021.685189 (PMC8222996; doi:10.3389/fpls.2021.685189)

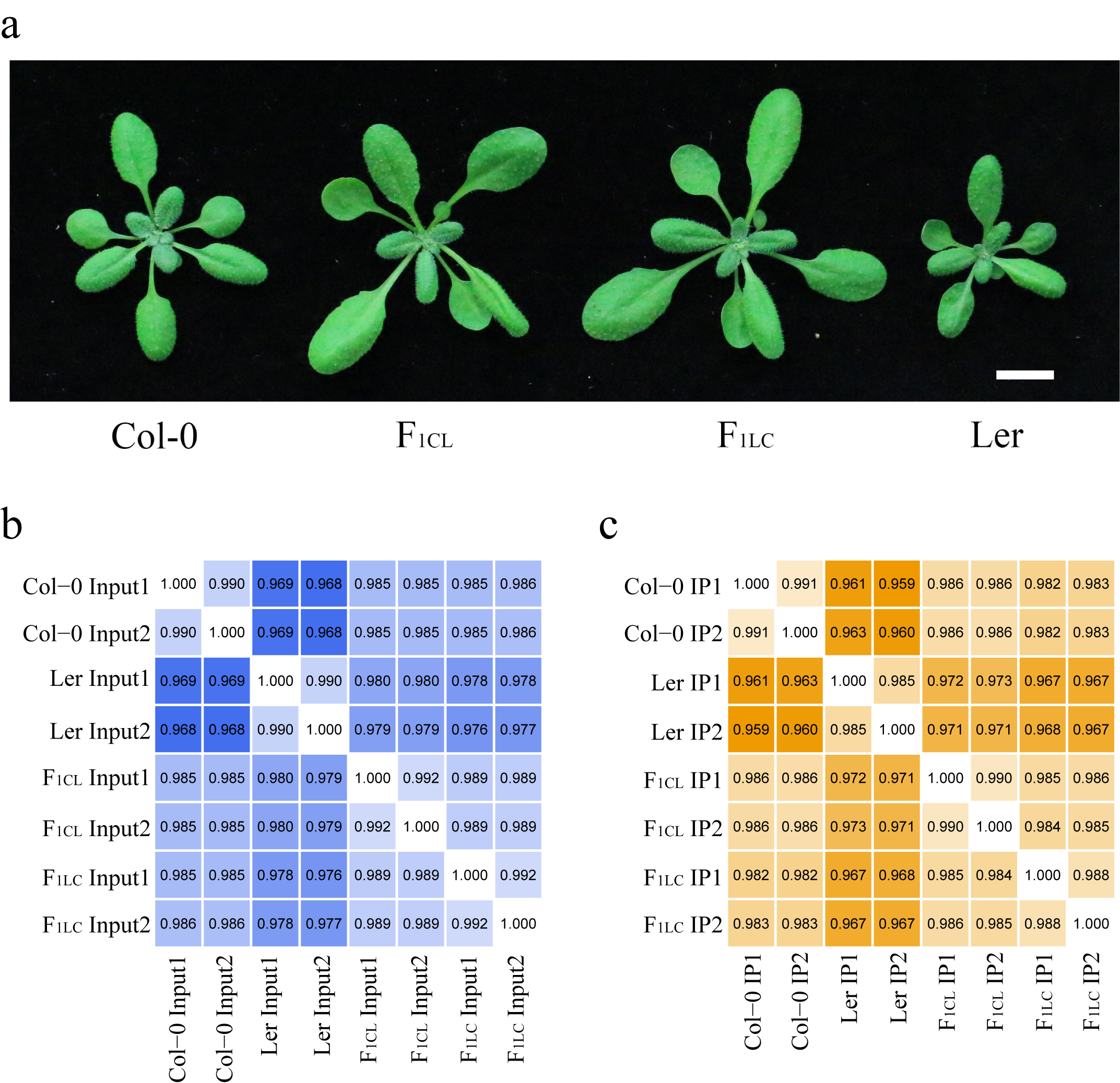

Supplement: Supplementary Figure 1 — Phenotypes of Arabidopsis lines and quality of sequencing data. (A) The biomass vigor of both F1 hybrids is higher than that of Col-0 and Ler. Scale bar = 10 mm. Spearman correlations between two biological replicates of input (B) mRNA-seq and (C) m6A-seq in Col-0, Ler, F1CL, and F1LC.. [file Image_1.TIF]

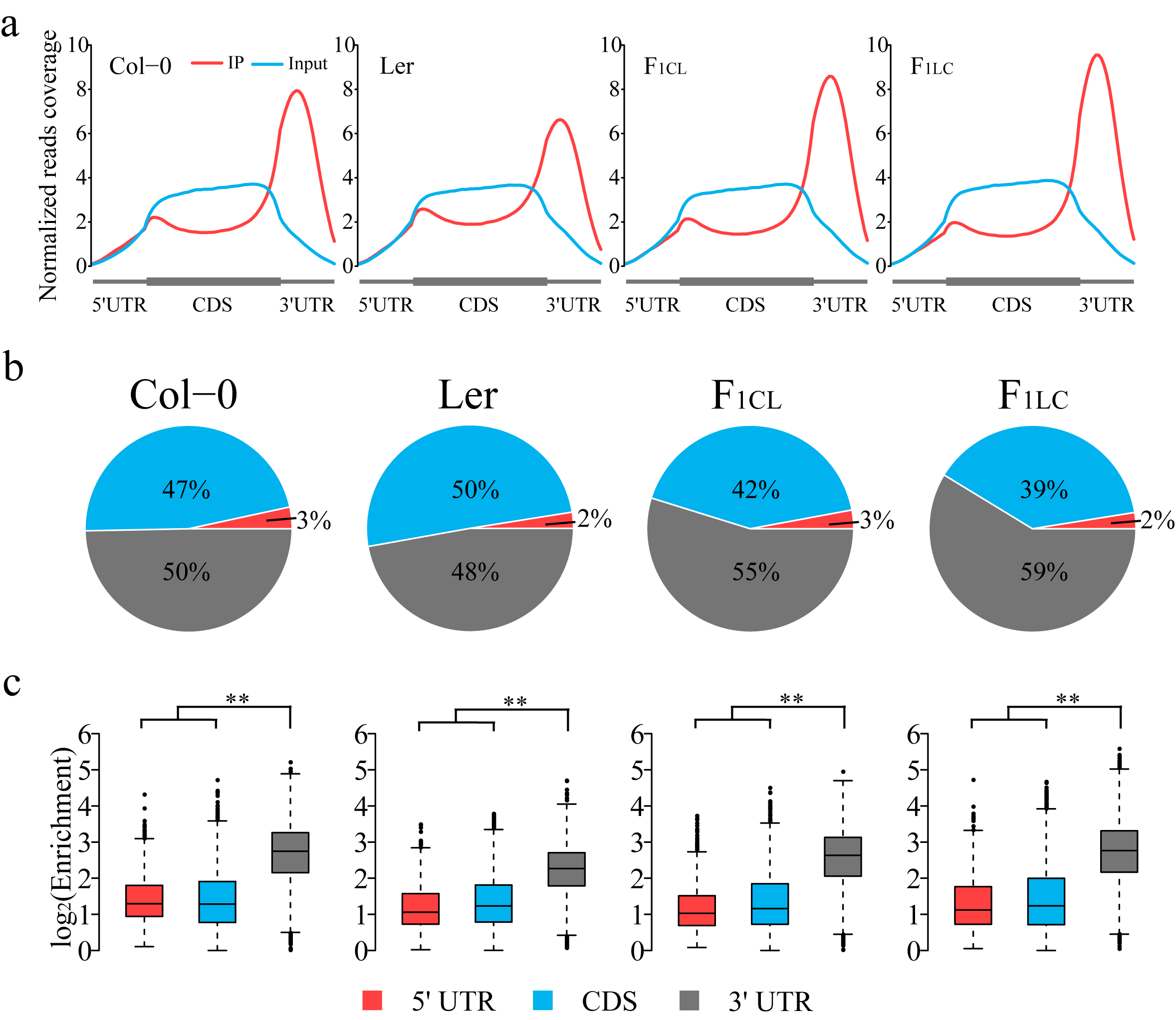

Supplement: Supplementary Figure 2 — Global pattern of m6A peaks using the Ler genome sequence as a reference. (A) Coverage of normalized reads along transcripts. Each transcript is divided into three non-overlapping features: 5' UTR, CDS, and 3' UTR. (B) Distribution of m6A peaks in transcript features of parents and hybrids. (C) Relative enrichment of m6A peaks of each transcript feature. Enrichment = Normalized m6A-seq read number divided by normalized input reads of each peak. **p < 2.2e−16, Wilcoxon rank-sum test. [file Image_2.TIF]

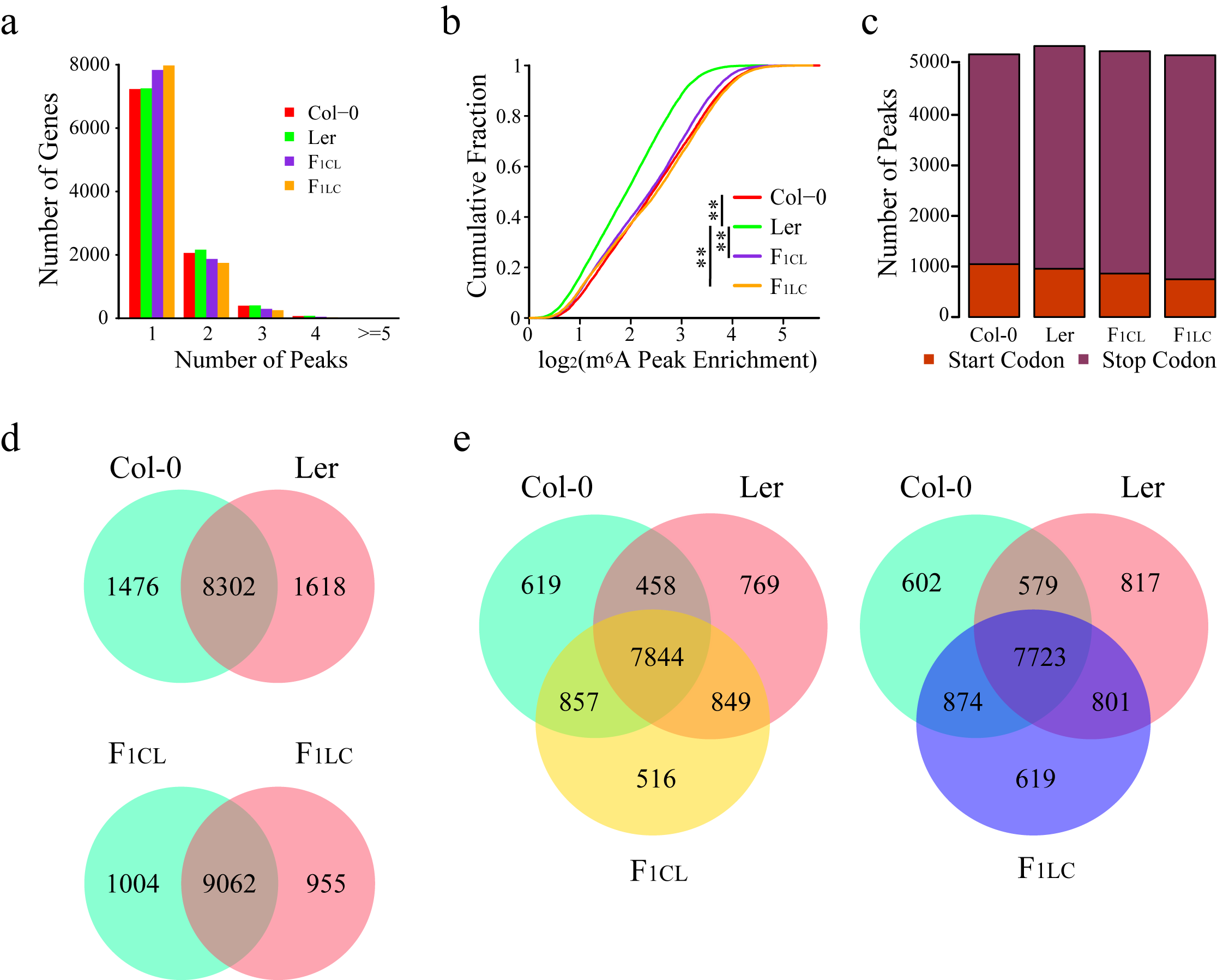

Supplement: Supplementary Figure 3 — Features of m6A modifications among the parent lines and F1 hybrids. (A) Number of peaks on transcripts. (B) Cumulative plot of m6A methylation enrichment in Col-0, Ler, F1CL, and F1LC. **p < 2.2e−16, Wilcoxon rank-sum test. (C) Number of m6A peaks located at the start codon and stop codon of transcripts. (D,E) Number of shared genes containing m6A peaks between accessions. [file Image_3.TIF]

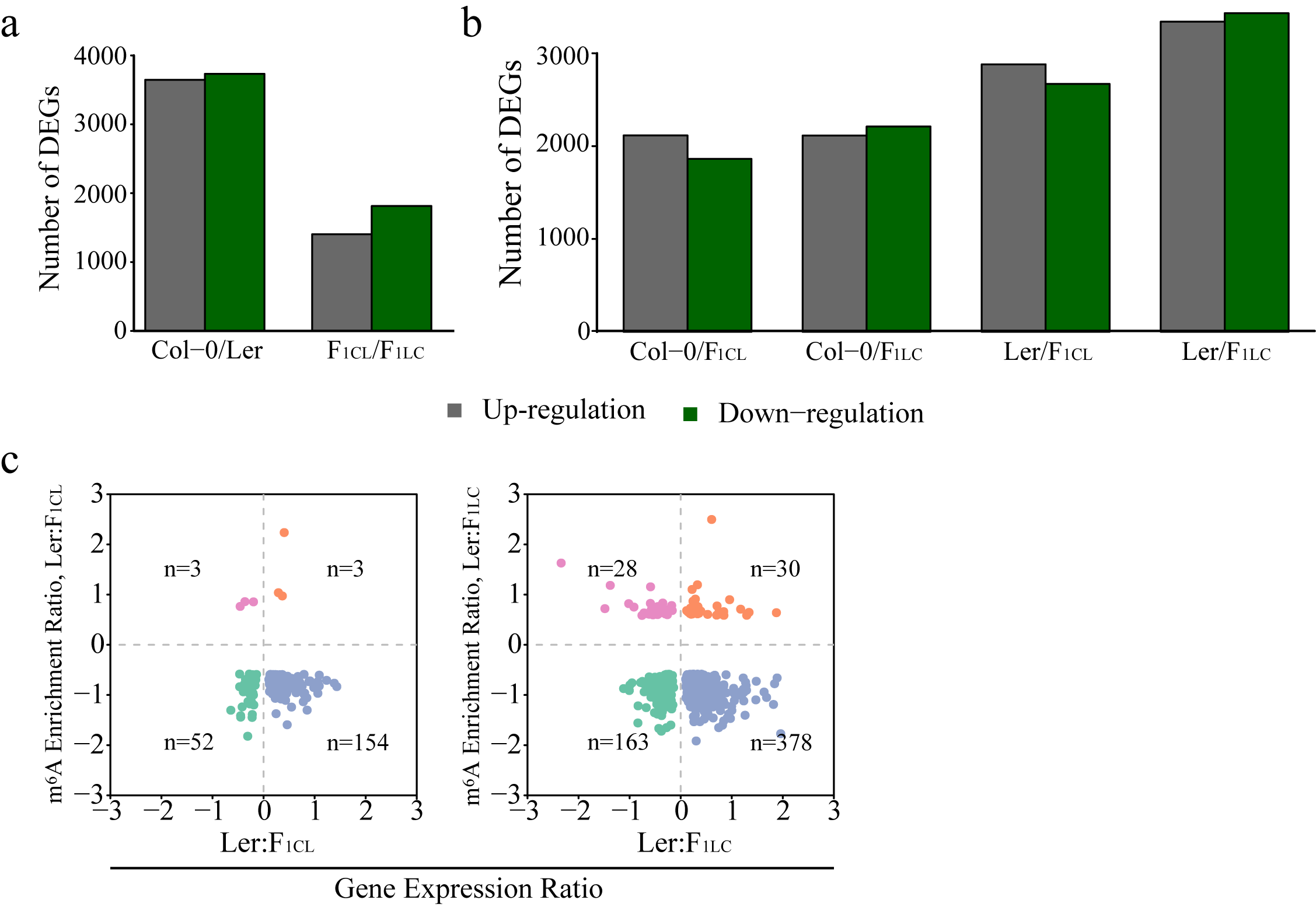

Supplement: Supplementary Figure 4 — Diagram of the relationship between m6A methylation level and transcript abundance. (A) Number of DEGs between parents and hybrids. (B) Number of up- or downregulated DEGs in comparisons of parents/hybrids. (C) Scatter plot of DMG-DEGs between accessions showing the relationship of m6A modification and transcript abundance. DMG-DEG indicates DEGs overlapping with DMGs (genes with differentially m6A-methylated peaks). For example, the m6A enrichment ratio of Ler: F1CL is calculated as log2 (enrichment of Ler/enrichment of F1CL) of m6A peaks. The gene expression ratio of Ler: F1CL is calculated as log2 (FPKM of Ler/FPKM of F1CL) of transcripts. n indicates the number of DMG-DEGs in each quadrant. Gene m6A enrichment is calculated by normalized m6A-seq reads number divided by normalized input reads of peaks within the transcript, and gene expression is indicated by the FPKM of the input RNA-seq data. [file Image_4.TIF]

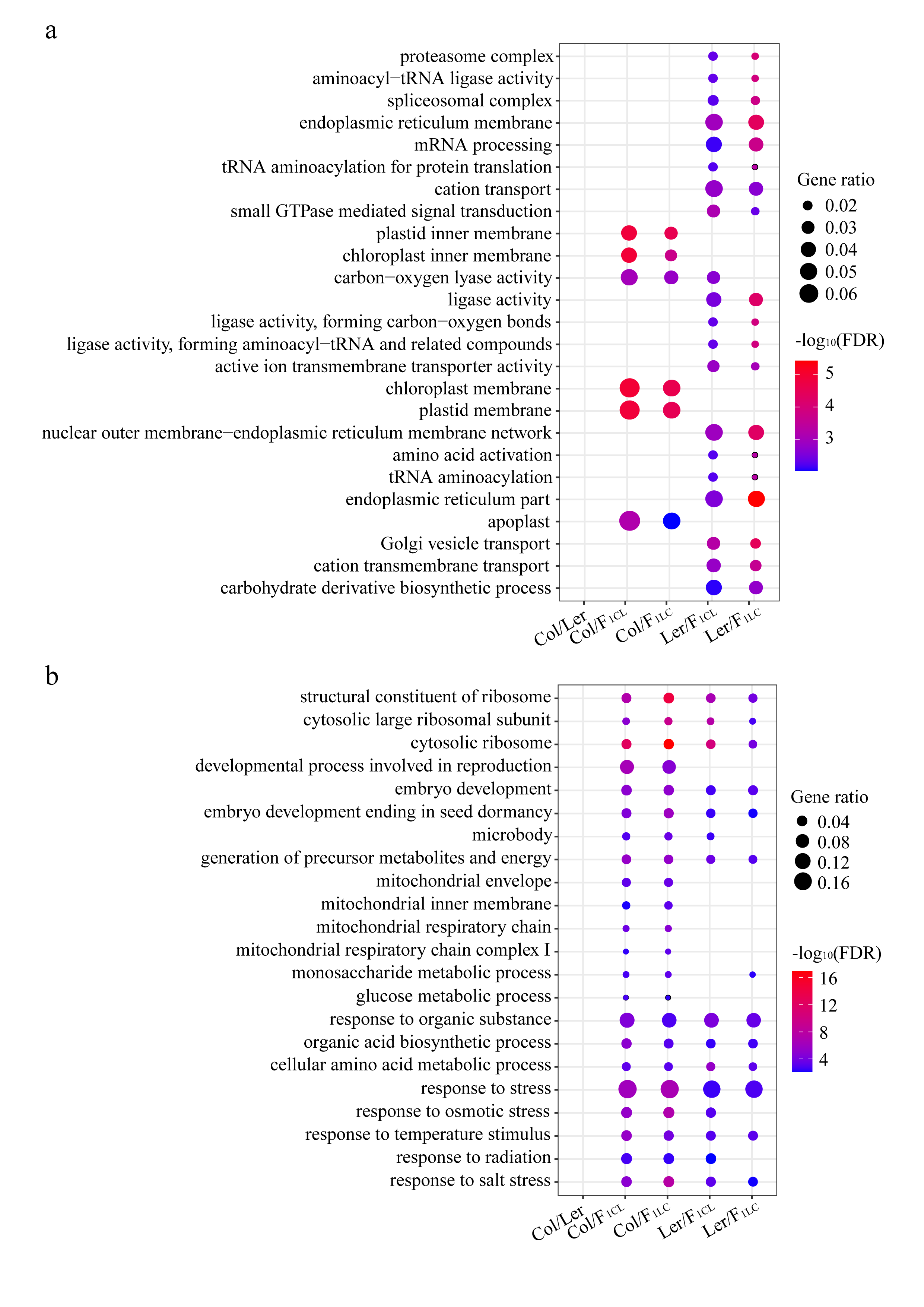

Supplement: Supplementary Figure 5 — Enriched biological functions of differentially m6A-methylated genes. (A) Enriched GO terms of genes associated with differentially m6A-methylated peaks (DMPs) that are not DEGs in comparisons of parents/hybrids. (B) Enriched GO terms of DEGs not associated with differentially m6A-methylated peaks (DMPs) in comparisons of parents/hybrids. Only some of the enriched GO terms enriched in the comparisons between parents/hybrids rather than Col/Ler are shown in the figure. All the GO terms are listed in Supplementary Table 4. [file Image_5.TIF]
