# Supplementary material for: Transcriptome-Wide Analysis of RNA m6A Methylation and Gene Expression Changes Among Two Arabidopsis Ecotypes and Their Reciprocal Hybrids
Source: Front Plant Sci. 2021 Jun 10;12:685189. doi: 10.3389/fpls.2021.685189 (PMC8222996; doi:10.3389/fpls.2021.685189)
Supplement: Supplementary file 13 [file Data_Sheet_1.ZIP › Readme.pdf]

Scripts used in study

script 1: calculate_tx_cov.py ....

script 2: get_reads_at_each_base_of_peaks.py ....

script 3: get_peak_summit.py..

script 4: assign_peak_to_utr_or_cds.py ....

script 5: get_reads_in_peak_region.py...

script 6: get_reads_of_alleles_by_informative_snps.py..

calculate_tx_cov.py

Descirption:

Calculate the reads along transcripts.

Usage:

python calculate_tx_cov.py –i <in.bam> -r <utr_cds_locaction_file> -n <n1,n2,n3> -o <output>

Arguments and options:

-I <string>, bam file of unique mapped reads sorted by position.

-r <string>,the file contains UTR and CDS location information.

-n <int>, bins for 5' UTR, CDS and 3' UTR.

-o <output>, output file.

The format of utr and cds location file is as below:

Chrom	Gene	Start	End	Strand	5' UTR	CDS	3' UTR
Chr1	AT1G01030	11649	13714	-	12941,13173,<br>13335,13714,	11864,12940,	11649,11863,

get_reads_in_peak_region.py

Descirption:

Calculate reads covered each peak region.

Usage:

python get_reads_in_peak_region.py <peak_region> <bam> <mapped_reads> <output>

Arguments:

<bam> is position sorted bam file.

<mapped_reads> is the number of unique reads mapped to the reference.

The format of peak region file:

Chrom	Peak	Start	End
Chr1	AT1G01140_1	64264	13714
Chr1	AT1G01430_1	158277	158426

get_reads_at_each_base_of_peaks.py

Descirption

Calculate the number of reads at each base of peak region.

Usage:

python get_reads_on_each_base_of_peaks.py <peak_region> <bam> <mapping_reads> <output>

*the description of file format is the same as above.

get_peak_summit.py

Descirption:
Identify peak summit by substracting IP reads from input reads.
Usage:
python get_peak_summit.py <peak_region> <input1_reads> <input2_reads> <ip1_reads> <ip2_reads> <output>
Arguments:
<peak_region>, the format is as above.
<input1_reads>, <input2_reads>, <ip1_reads>, <ip2_reads> are output files of get_reads_on_each_base_of_peaks.py, which have the number of reads covered at each base of peak region.

assign_peak_to_utr_or_cds.py

Descirption
Assign peak to utr or cds region according to its summit.
Usage:
python assign_peak_to_utr_or_cds.py <peak_summit> <utr_cds_locaction_file> <output1> <output2>
Arguments:
<peak_summit>, output file of get_peak_summit.py.
<utr_cds_locaction_file>, the file contains UTR and CDS location information. Described above.
<output1>, the number of peaks assigned to UTR or CDS.
<output2>, the details of annotation of each peak.

get_reads_of_alleles_by_informative_snps.py

Descirption:
Count reads on allelic transcripts or peaks by SNPs.
Usage:
python get_reads_of_alleles_by_informative_snps.py <peak_region> <snp_list> <bam1> <bam2> <output>
Arguments:
<peak_region>, decribed as above.
<bam1, bam2>, bam file sorted by position.
<snp_list>, the file contains SNPs that identified by comparing Col-0 and Ler reference genomes. It have four columns, each column represents chromsome, position, reference and SNP, respectively. For example, Chr1 105064 C T.
